# Association between premature ovarian insufficiency and gut microbiota

**DOI:** 10.1186/s12884-021-03855-w

**Published:** 2021-06-05

**Authors:** Jiaman Wu, Yuanyuan Zhuo, Yulei Liu, Yan Chen, Yan Ning, Jilong Yao

**Affiliations:** 1 Affiliated Shenzhen Maternity&Child Healthcare Hospital, Southern Medical University, No. 3012, Fuqiang Road, Futian District, Shenzhen City, 518000 Guangdong Province China; 2 Shenzhen Traditional Chinese Medicine Hospital, Shenzhen, 518033 China

**Keywords:** Premature ovarian insufficiency, Gut microbiota, Sex hormones, 16S rRNA sequencing

## Abstract

**Background:**

Premature ovarian insufficiency (POI) is characterized by impairment of ovarian function on a continuum before the age of 40 years. POI is affected by multiple factors. Considering new insights from recent gut microbiome studies, this study aimed to investigate the relationship between gut microbial community structure and POI.

**Methods:**

Subjects were recruited at the Shenzhen Maternity & Child Healthcare Hospital. Fecal microbial community profiles of healthy women (*n* = 18), women with POI (*n* = 35) were analyzed using 16S rRNA gene sequencing based on Illumina NovaSeq platform.

**Results:**

Compared to the controls, the serum levels of FSH, LH, T and FSH/LH ratio significantly increased in women with POI, whereas E2 and AMH decreased significantly. Higher weighted UniFrac value was observed in POI women compared with healthy women. Phylum *Firmicutes,* genera *Bulleidia* and *Faecalibacterium* were more abundant in healthy women, while phylum *Bacteroidetes,* genera *Butyricimonas, Dorea, Lachnobacterium* and *Sutterella* enriched significantly in women with POI. Moreover, these alterations of the gut microbiome in women with POI were closely related to FSH, LH, E2, AMH level and FSH/LH ratio.

**Conclusions:**

Women with POI had altered microbial profiles in their gut microbiome, which were associated with serum hormones levels. These results will shed a new light on the pathogenesis and treatment for POI.

## Background

Premature ovarian insufficiency (POI) is an ovarian insufficiency syndrome before the age of 40 years affecting approximately 1–2% women [26, 12]. It is characterized by a continuous decline in ovarian function, and resulting in an earlier cessation of menstruation than normal [12]. Women with POI are faced with increased risk of low chance of natural conception [4, 38], urogenital atrophy [34], decrease in bone mineral density [3], autoimmune and thyroid disease risk [11], cognitive dysfunction [18], shortened life expectancy [30], and cardiovascular disease [44, 15]. POI is a multifactorial disease [33]. Spontaneous POI is associated with genetic defects, autoimmunue diseases, enzyme deficiency and environmental factors, and iatrogenic POI occurs mainly due to surgical intervention, chemotherapy and radiotherapy [18, 33].

There is ample evidence indicating that ovary is damaged by autoimmunity through alteration of T-cell subsets, T-cell-mediated injury, increasing of autoantibody-producing B-cells, decreasing of effector suppressor/cytotoxic lymphocytes, and decreasing of natural killer cells [11]. It supports that antoimmune etiology exists in POI based on the presence of lymphocytic oophoritis, association with autoimmune disorders, and autoantibodies [11, 21]. A number of studies have demonstrated that gut microbiome play a key role in the autoimmune process [45]. Such as the peptides generated by gut microbiota could induce immune cells to become autoreactive and crossactivated [45]. Moreover, the dysbiosis of gut microbiome not only can affect the activation of B lymphocytes and the production of autoantibodies, but also induce the aberrant activation of innate immune cells, which leads to the upregulation of proinflammatory cytokines [45]. In addition, increasing evidences have strongly suggested that gut microbiome play an important role in POI-associated symptoms, including autoimmune dysfunction [39, 8], bone health [20, 29], cognitive and neurological health [28, 14]. Gut microbiota and its metabolites also have the ability to regulate inflammation pathway activation, brain-gut peptide secretion and the destruction of islet β-cell [46, 22]. All these studies indicate a relationship may exist between the gut microbiome and POI.

In order to study the community profile of gut microbiome in women with POI, and how the changes of gut microbiota correlated with the sex hormones, 35 women with POI and 18 healthy women were recruited in this study. Sequencing of the V3-V4 regions of 16S rRNA gene in fecal samples was performed to reveal the substantial differences of gut microbiota between the POI subjects and controls.

## Methods

### Study cohort

A total of 35 women with spontaneous POI and 18 healthy women, aged 24 to 40 years, were recruited at the Shenzhen Maternity&Child Healthcare Hospital from August 2019 to September 2019. Spontaneous POI was diagnosed and assessed according to the previously reported [17], primary or secondary amenorrhea for at least 4 months before 40 years, and with at least two instances of serum follicle-stimulating hormone (FSH) levels exceeded 40 IU/L with an interval of 4–6 weeks. All the control women had normal ovarian function, without history of menstrual dysfunction and infertility, with regular menstruation and normal levels of FSH (< 10 IU/L). Participants were excluded if with following situations: non-46-XX karyotype, POI with family history, pregnancy, tumor, chronic diarrhea, autoimmune diseases, use of antibiotics/medications with the preceding 3 months, pelvic surgery, gastrointestinal disease, active infections, body mass index < 18.5 or > 23.9 kg/m^2^, smoking and chemo/radiotherapy treatment. The study protocol was approved by the ethics committee of Shenzhen Maternity&Child Healthcare Hospital. Written informed consents were obtained from all participants prior to enrollment. And clinical characteristics were extracted from the health records.

### Sampling

All participants were examined in the morning after > 8 h fasting. Fecal samples were collected using empty stool collection tubes with an inbuilt sterile swab. Samples were stored at − 80 °C until further analysis.

### DNA extraction and sequencing

DNA was extracted from fecal samples using QIAamp DNA stool mini kit (Qiagen, Germany) according to the manufacturer’s instructions. PCR amplification was conducted using 338F forward primer 5′-ACTCCTACGGGAGGCAGCAG-3′ and 806R reverse primer 5′-GGACTACHVGGGTWTCTAAT-3′ targeting the variable V3-V4 regions of 16S rRNA gene. All samples were pooled equally and sequenced on an Illumina NovaSeq 6000 machine with 2 × 250 flow cell. Raw sequencing data in this study were deposited into the NCBI’s Sequence Read Archive database (SRA BioProject ID PRJNA615330).

### Sequencing data analysis

Sequencing reads were assigned to each sample based on dual-index barcodes using custom Perl script. Reads were processed using the bioinformatics software package QIIME2 (version 2019.10). Firstly, the reads were imported to a QIIME2 artifact with command “qiime tool import”. Then the reads were denoised with command “qiime data 2 denoise-paired” to exclude chimeric sequences and phiX sequences. Next, taxonomy was assigned with command “qiime2 feature-classifier classify-sklearn” against Greengenes (13_8 revision) database. Meanwhile, Shannon index and weighted UniFrac distance were generated with command “qiime phylogeny align-to-tree-mafft-fasttree” and “qiime diversity core-metrics-phylogenetic” at a sample depth of 1000. Principal coordinate analysis (PCoA) based on weighted UniFrac distances were also calculated.

### Statistical analysis

Permutational multivariate analysis of variance (PERMANOVA) was performed on weighted UniFrac distance to investigate the differences of microbial community structure between POI group and healthy control group using package vegan (999 permutations) in R software.

Statistical calculations were performed using R software, and *P* value < 0.05 was considered significantly different. Normality test was conducted using Shapiro-Wilk test. Unpaired *t*-tests were used for comparisons of clinical characteristics, indices of diversity between the two groups. All these continuous data were expressed as mean value ± standard deviation (SD). While Wilcoxon Rank Sum tests were used for taxa comparisons at the phylum and genus level. Partial correlation analysis was used to investigate the relationships between microbes and clinical characteristics in R packages “ggm” and “psych”.

## Results

### Study subject characteristics

Study subjects are all Han Chinese and live in Shenzhen City. Their characteristics were summarized in Table 1. All participants aged from 24 to 40 years old (average: 34.6 years old), accompanied with body mass index (BMI) ranging from 18.6 to 23.9 kg/m^2^ (average: 21.2). Statistical analysis demonstrated that there were no differences at age, progesterone (P), prolactin (PRL) and glucose (GLU) between women with and without POI. In addition, women with POI had significantly higher levels of BMI, FSH, luteinizing hormone (LH), testosterone (T) and FSH/LH ratio, but significantly lower levels of estradiol (E2) and anti-Mullerian hormone (AMH), compared to healthy control women.

**Table 1:** Demographic and clinical characteristics of the two groups

Characteristic	POI = 35	NG = 18	***P*** value
Age (years)	35.23 ± 4.62	33.5 ± 4.05	0.19
BMI (kg/m^2^)	21.5 ± 1.35	20.54 ± 1.61	0.02*
FSH (mIU/mL)	45.60 ± 28.77	5.39 ± 1.74	< 0.01**
LH (mIU/mL)	15.79 ± 9.02	4.04 ± 1.07	< 0.01**
E2 (pg/L)	30.71 ± 11.7	54.56 ± 9.0	< 0.01**
P (nmol/L)	0.48 ± 0.33	0.35 ± 0.14	0.12
T (nmol/L)	0.43 ± 0.22	0.31 ± 0.11	0.03*
PRL (nmol/L)	14.37 ± 7.12	11.49 ± 4.57	0.13
AMH (ng/mL)	0.49 ± 0.35	3.94 ± 2.04	< 0.01**
FSH/LH (ratio)	2.88 ± 0.68	1.32 ± 0.18	< 0.01**
GLU (nmol/L)	5.07 ± 0.41	4.99 ± 0.32	0.48

*BMI* body mass index, *FSH* follicle-stimulating hormone, *LH* luteinizing hormone, *E2* estradiol, *P* progesterone, *T* testosterone, *PRL* prolactin, *AMH* anti-Mullerian hormone, *GLU* glucose

*P <0.05; **P < 0.01

### Overall community structure of POI gut microbiome

Sequencing was performed on the V3-V4 regions of 16S rRNA to evaluate the community structure of gut microbiota in women with and without POI. In total, 3,163,487 usable reads (59,688 ± 11,095 reads per sample) were obtained from all 53 samples, and the mean and median sequence lengths were 411 and 410 bp separately. The number of reads analyzed did not differ between POI and control samples (60,943 ± 11,393 versus 57,247 ± 10,326, *P* = 0.25), indicating comparable and adequate sequencing coverage.

**Fig. 1:**
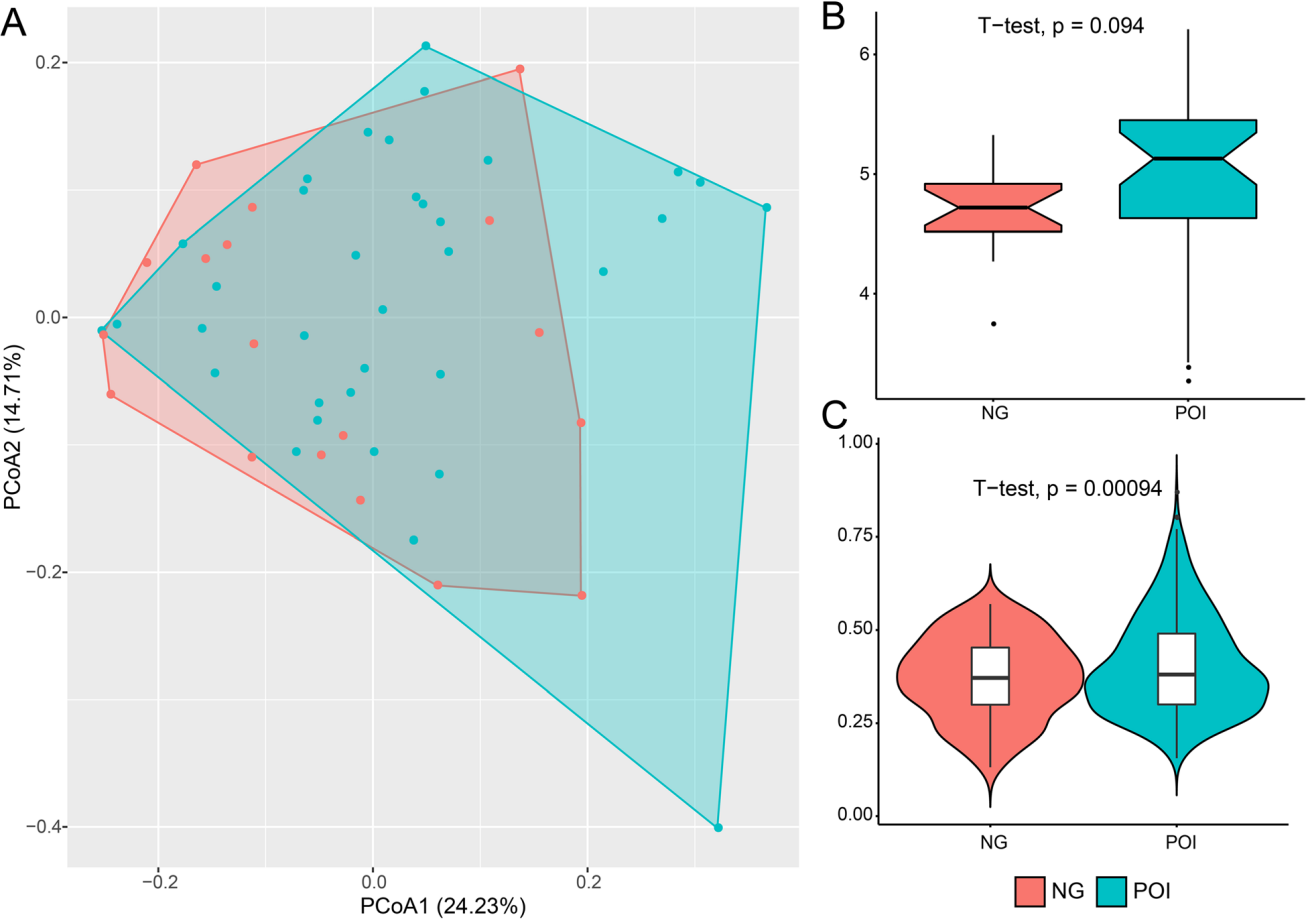
Overall structural differentiation of gut microbiota between the two groups. POI represented women with premature ovarian insufficiency, NG represented healthy control women. **a** PCoA analysis plot based on weight UniFrac value. **b** Shannon index between the two groups. **c** Weighted UniFrac value between the two groups

To explore the dissimilarity of gut microbiota between the two groups, PCoA analysis was performed based on the weighted UniFrac distance. The results showed the subjects of the two groups did not separate (Fig. 1a) (*P* = 0.16, PERMANOVA analysis with 999 permutations). Further, POI subjects exhibited a higher Shannon index without significant difference between the two groups (4.97 ± 0.74 versus 4.71 ± 0.39, *P* = 0.09) (Fig. 1b). The average weighted UniFrac value within subjects of POI group was significantly higher than control group (0.41 ± 0.13 versus 0.37 ± 0.11, *P* < 0.01) (Fig. 1c).

### Characterizing the gut microbiome in POI group

**Fig. 2:**
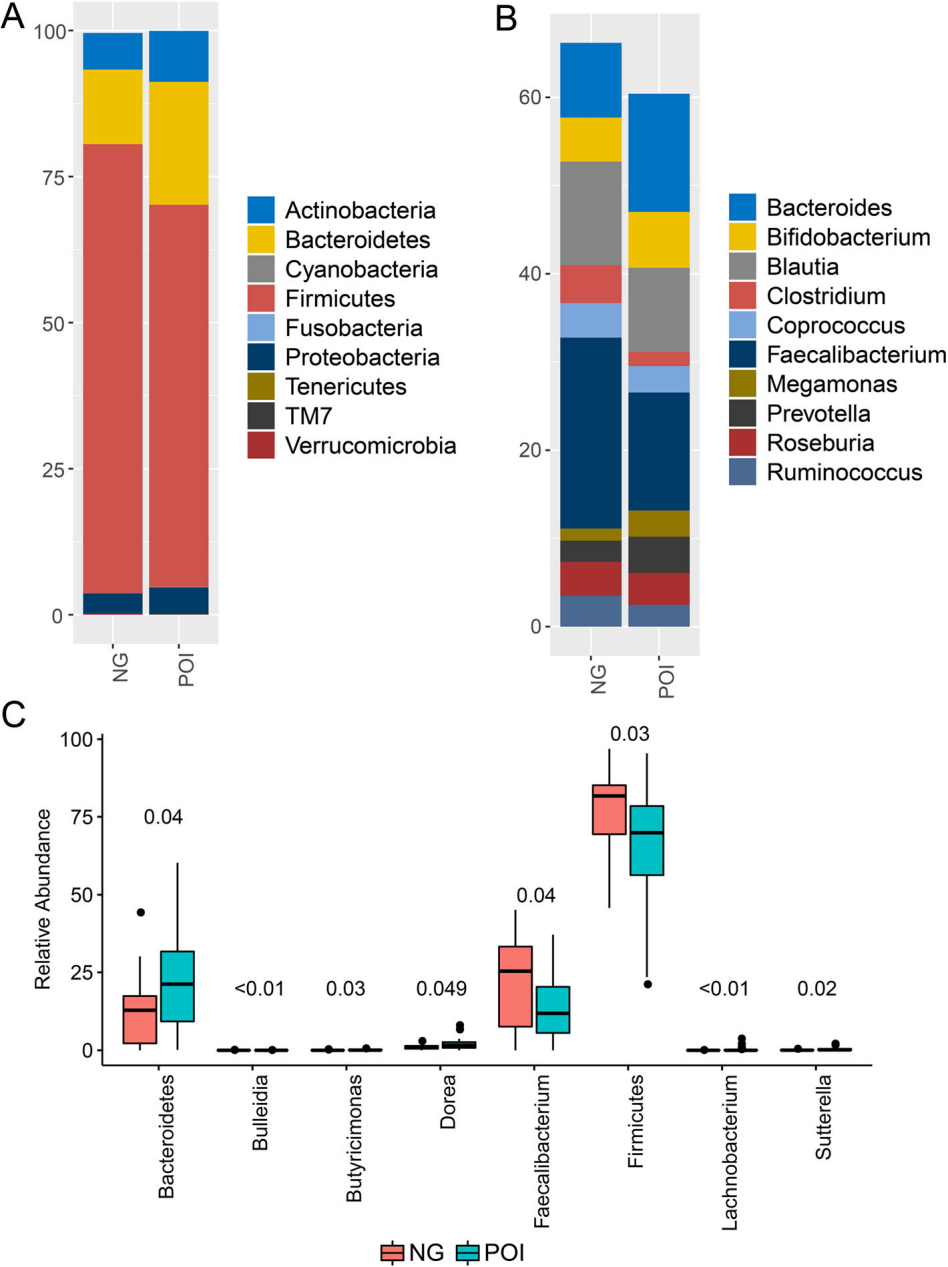
Microbial community profiles of gut microbiota between POI and control group. POI represented women with premature ovarian insufficiency, NG represented healthy control women. **a** Relative abundances of the dominant phylum. **b** Relative abundances of the top 10 genera according to the relative abundance. **c** Significant different and important microbes between POI and control group

Gut microbiome communities were dominated by phyla *Firmicutes*, *Bacteroidetes, Actinobacteria* and *Proteobacteria* in both POI and control groups (Fig. 2a). And *Firmicutes* was the predominant microbe, accounting for 65.35% ± 18.71 and 76.83% ± 14.90% in POI and control group, respectively. The top 10 abundant genera in both two groups were *Bacteroides*, *Bifidobacterium*, *Blautia*, *Clostridium*, *Coprococcus*, *Faecalibacterium*, *Megamonas*, *Prevotella*, *Roseburia* and *Ruminococcus* (Fig. 2b). Compared to the control group, *Bacteroides* (13.42% ± 11.06% versus 8.45% ± 10.14%), *Bifidobacterium* (6.30% ± 11.76% versus 5.0% ± 8.84%), *Megamonas* (2.97% ± 12.90% versus 1.37% ± 4.61%), *Prevotella* (4.09% ± 9.90% versus 2.37% ± 4.62%) increased, whereas *Blautia* (9.60% ± 7.37% versus 11.72% ± 7.90%), *Clostridium* (1.61% ± 2.13% versus 4.31% ± 7.99%), *Coprococcus* (3.0% ± 2.93% versus 3.91% ± 4.12%), *Faecalibacterium* (13.35% ± 10.39% versus 21.65% ± 14.80%), *Roseburia* (3.63% ± 4.72% versus 3.84% ± 3.98%) and *Ruminococcus* (3.73% ± 3.46% versus 4.75% ± 5.90%) decreased in POI group.

Through Wilcoxon Rank Sum tests (Fig. 2c), phylum *Bacteroidetes* (21.10% ± 14.01% versus 12.72% ± 11.96%, *P* = 0.04), genera *Butyricimonas* (0.12% ± 0.16% versus 0.04% ± 0.08%, *P* = 0.03)*, Dorea* (1.91% ± 1.72% versus 1.02% ± 0.83%, *P* = 0.049)*, Lachnobacterium* (0.26% ± 0.72% versus 0.0011% ± 0.0047%, *P* = 0.007) and *Sutterella* (0.34% ± 0.46% versus 0.08% ± 0.11%, *P* = 0.02) significantly increased in POI women, compared to control group. Whereas phylum *Firmicutes* (*P* = 0.03), genera *Bulleidia* (0.0006% ± 0.0033% versus 0.0094% ± 0.021%, *P* = 0.007) and *Faecalibacterium* (*P* = 0.04) significantly decreased. Moreover, *Bacteroidetes/Firmicutes* ratio (0.41 ± 0.44 versus 0.20 ± 0.24, *P* = 0.03) in POI group was significantly higher than control group.

### Association between differential gut microbiota and clinical characteristics

**Fig. 3:**
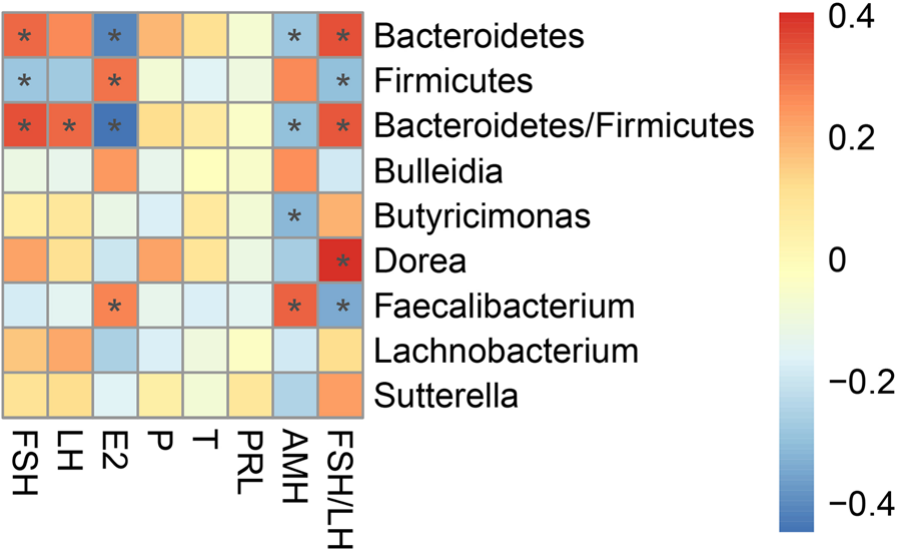
Associations between gut microbiota and serum hormones. The star indicate the significant correlation at 5% level

Pearson correlation analysis was performed after adjusting for BMI to evaluate the association between the differential microbes and serum hormones (Fig. 3). The results showed that E2 level was significantly negatively correlated with relative proportion of *Bacteroidetes* (*R* = − 0.41, *P* = 0.002) and *Bacteroidetes/Firmicutes* ratio (*R* = − 0.45, *P* < 0.01), while positively correlated with *Firmicutes* (*R* = 0.30, *P* = 0.03) and *Faecalibacterium* (*R* = 0.28, *P* = 0.047). FSH was significantly positively correlated with *Bacteroidetes* (*R* = 0.31, *P* = 0.02)*, Bacteroidetes/Firmicutes* ratio (*R* = 0.35, *P* = 0.01), and negatively correlated with *Firmicutes* (*R* = − 0.28, *P* = 0.04). LH was significantly positively correlated with *Bacteroidetes/Firmicutes* ratio (*R* = 0.31, *P* = 0.02). FSH/LH ratio was significantly positively correlated with the relative proportion of *Bacteroidetes* (*R* = 0.35, *P* = 0.01), *Bacteroidetes/Firmicutes* ratio (*R* = 0.34, *P* = 0.01), *Dorea* (*R* = 0.40, *P* < 0.01), and negatively correlated with the relative proportion of *Firmicutes* (*R* = − 0.30, *P* = 0.03) and *Faecalibacterium* (*R* = − 0.34, *P* = 0.01). AMH level was significantly correlated with the relative proportion of *Bacteroidetes* (*R* = − 0.28, *P* = 0.04), *Bacteroidetes/Firmicutes* ratio (*R* = − 0.30, *P* = 0.03), *Butyricimonas* (*R* = − 0.31, *P* = 0.02) and *Faecalibacterium* (*R* = 0.33, *P* = 0.02).

## Discussion

This study aimed to reveal the overall composition of gut microbiota in women with POI. As 15 to 30% of POI occurrences are considered to be familial [12]. To rule out genetic influences, all subjects recruited in this study were without blood relationship. Besides genetic factor, autoimmunity is an important etiology for POI, and gut microbiome is important for the development and maturation of the host immune system. In this study, the results showed that all recruited subjects composed primarily of phyla *Actinobacteria*, *Bacteriodetes*, *Firmicutes* and *Proteobacteria*. The sum relative abundance of *Bacteriodetes* and *Firmicutes* accounted for more than 90%, which was consistent with previous studies [42, 1]. A balance between *Bacteriodetes* and *Firmicutes* is important to maintain intestinal homeostasis [1], while a significant higher *Bacteriodetes/Firmicutes* ratio was observed in POI subjects compared to control subjects. Furthermore, the abundant genera were *Bacteroides*, *Bifidobacterium*, *Blautia*, *Clostridium*, *Coprococcus*, *Faecalibacterium*, *Megamonas*, *Prevotella*, *Roseburia* and *Ruminococcus*. Most of them play important roles in the maintenance of host gastrointestinal homeostasis and health [37, 25]. Notably, *Bacteroides fragilis* produce some peptides similar to type II collagen to induce crossreactive responses and to promote Th17 responses [45]. Similarly, some peptides produced by *Bacteroides thetaiotaomicron* and *Roseburia intestinalis* show similarity with human Ro60 and β2-glycoprotein I, which can trigger lupus-like symptoms [36, 16]. *Bifidobacterium adolescentis* can induce and promote Th17 responses in intestine [40]. *Prevotella copri* also can express antigens, and promote Th17 responses [27]. While the relative abundances of genera *Bacteroides, Bifidobacterium* and *Prevotella* all increased in POI group in this study. Moreover, genera *Bifidobacterium*, *Blautia, Clostridium, Faecalibacterium, Roseburia* and *Ruminococcus* can produce short chain fatty acids (SCFAs) in human gut, such as acetate, butyrate and succinate and so on [31, 35]. SCFAs not only have anti-inflammatory and immunomodulatory properties through modulating Treg/Th17 cell balance, but also can influence psychological function and cognitive processes [35, 7]. Butyrate was reported to alleviate antibody-induced arthritis in a natural killer T cell-dependent manner [23]. While the relative abundances of *Blautia, Clostridium*, *Faecalibacterium, Roseburia* and *Ruminococcus* presented decrease trends, especially *Faecalibacterium* decreased significantly in POI women in this study. All theses changes of gut microbiome in POI group might induce immunomodulating activity through certain bacterial strains and their metabolites, which might be related to autoimmunity, then affects the development of POI.

Further, the gut microbiome is also found to be related to POI-associated autoimmune disorders [8]. In this study, there were significant decrease of *Firmicutes*, and significant increase of *Bacteroidetes* and *Bacteroidetes/Firmicutes* ratio in POI women. It was consistent with the microbial community structure of type 1 diabetes and systemic lupus erythematosus patients, with a low proportion of *Firmicutes,* high proportion of *Bacteroidetes* and *Bacteroidetes/Firmicutes* ratio [10, 19]. Moreover, genera *Dorea* and *Sutterella* were more abundant in POI group. Increased proportion of *Dorea* is related to multiple multiple sclerosis, an autoimmune condition [6]. *Sutterella* is a mildly pro-inflammatory genus, and its elevated level is associated with cognitive function [43, 41]. As a SCFAs producing member with anti-inflammatory properties, *Faecalibacterium* decreased significantly in POI group in this study, its low proportion is also related to multiple sclerosis [5]. Butyrate producer *Butyricimonas* [9] increased significantly in POI group in this study. On the contrary, a low abundance of *Butyricimonas* was observed in multiple sclerosis fecal samples [13], that might be due to different species under *Butyricimonas*. Type 1 diabetes, systemic lupus erythematousus and multiple sclerosis, and cognitive dysfunction are all closely related to POI. Thus, the alterations of gut microbiome observed in this study might be related to the development of POI through affecting on the POI-associated diseases.

The gut microbiome has been shown to play an important part in impacting estrogen level through the secretion of β-glucuronidase, which could deconjugate estrogen and affect related physiological process [32, 1]. In this study, significant lower level of E2 was observed in POI subjects, and it was significantly correlated with the proportion of Bacteroidetes, Firmicutes and Faecalibacterium by adjusting for BMI. And the FSH, LH and AMH levels were also related to some microbes. It indicated that the altered gut microbiota of POI was associated with the sex hormones. Accumulating researches indicates that estrogen regulate glucose and lipid metabolism, bone formation and inflammatory response, its reduction can impair estrogen-dependent processes, triggering cardiovascular disease, osteoporosis and so on [2, 24]. These symptoms are all related to POI. Yet due to limited data in this study, the mechanism under the relations between these microbes and sex hormones is not clear.

POI leads to several complications, including decrease in bone mineral density, autoimmune, thyroid disease risk and cognitive dysfunction. This study not only revealed the association between gut microbiota and autoimmunity, but also the relationship between gut microbiota and these complications. These indicated that the dysbiosis of gut microbiota was related to the development of POI discussed above. However, limited sample size, participants from the same hospital and the observation of association but not causality, large sample size and multi-center are needed in the further studies. Moreover, metagenome sequencing, measurements of metabolites produced by gut microbiota, animal experiments should also be considered to explore the potential causal mechanism.

## Conclusions

In summary, POI may cause by autoimmune etiology, and the autoimmune process is affected by gut microbiome, so we may consider some relationship may exist between gut microbiome and POI.this study demonstrated an altered gut microbial pattern in women with POI against healthy controls, with an increase in Bacteroides, Bifidobacterium, Megamonas, Prevotella and a decrease in Blautia, Clostridium, Coprococcus, aecalibacterium, Roseburia and Ruminococcu. And we also found that these changes of microbes were closely related to serum hormones. This will help us to make a foundation for revealing the interaction between gut microbiota and POI certainly.

## Data Availability

The dataset supporting the conclusion of this article is available in the NCBI’s Sequence Read Archive database (SRA BioProject ID PRJNA615330).

## Abbreviations

BMI: Body mass index
FSH: Follicle-stimulating hormone
LH: Luteinizing hormone
E2: Estradiol
P: Progesterone
T: Testosterone
PRL: Prolactin
AMH: Anti-Mullerian hormone
GLU: Glucose
